# Hepatitis E Virus in Cambodia: Prevalence among the General Population and Complete Genome Sequence of Genotype 4

**DOI:** 10.1371/journal.pone.0136903

**Published:** 2015-08-28

**Authors:** Hiroko Yamada, Kazuaki Takahashi, Olline Lim, Somana Svay, Channarena Chuon, Sirany Hok, Son Huy Do, Mayumi Fujimoto, Tomoyuki Akita, Noboru Goto, Keiko Katayama, Masahiro Arai, Junko Tanaka

**Affiliations:** 1 Department of Epidemiology, Infectious Disease Control and Prevention, Institute of Biomedical and Health Sciences, Hiroshima University, Hiroshima, Japan; 2 Department of Medical Sciences, Toshiba General Hospital, Tokyo, Japan; 3 Ministry of Health, Phnom Penh, Cambodia; 4 Binh Thuan Medical College, Phan Thiet City, Binh Thuan Province, Vietnam; 5 Department of Management Studies, Graduate School of Social Sciences, Hiroshima University, Hiroshima, Japan; University of South Carolina School of Medicine, UNITED STATES

## Abstract

Hepatitis E virus (HEV) is a growing public health problem in many countries. In this study, we investigated HEV seroprevalence among the general population in the Siem Reap province, Cambodia, and performed HEV genetic analysis with the aim to develop an HEV prevention strategy. This seroepidemiological cross-sectional study conducted from 2010 to 2014 included 868 participants from four different locations in Siem Reap province, Cambodia. They answered questionnaires and provided blood samples for the analysis of hepatitis virus infections. Among the participants (360 men and 508 women; age range, 7–90 years), the prevalence of anti-HEV IgG was 18.4% (95% confidence interval: 15.9–21.0); HEV RNA was detected in two participants (0.23%) and was classified as genotype 3 and 4. Full-length genome of the genotype 4 isolate, CVS-Sie10, was sequenced; it contained 7,222 nucleotides and three ORFs and demonstrated high sequence identity with the swine China isolates swGX40 (95.57%), SS19 (94.37%), and swDQ (91.94%). Multivariate logistic regression analysis revealed that men, elderly people, and house workers were risk groups significantly associated with the positivity for anti-HEV IgG. This is the first report on the detection of HEV genotype 4 in humans in Cambodia and on the complete genome sequence of HEV genotype 4 from this country. Our study demonstrates that new HEV infection cases occur frequently among the general population in Cambodia, and effective preventive measures are required.

## Introduction

World Health Organization (WHO) statistics indicates that approximately 20 million people are hepatitis E virus (HEV)-infected, over 3 million have acute hepatitis E, and 70,000 die of hepatitis E every year worldwide [1, 2]. HEV is transmitted mainly through the fecal-oral route because of fecal contamination of drinking water; therefore, low sanitation standards increase the risk of HEV infection [1] which is a common cause of hepatitis outbreaks in the developing world [2]. In Cambodia, one of the developing countries in Asia, HEV infection can be an important health problem.

According to genome sequence, HEV has been classified into four genotypes; recently, new HEV genotype 5 has been identified in a wild boar in Japan [3]. HEV genotypes differ in their epidemiology and severity of infection. Genotype 1 is usually detected during hepatitis E outbreaks in developing countries in Asia, Africa, and South America; genotype 2 has been identified in Mexico, Chad, and Nigeria, while genotype 3 is more common in the developed countries, and genotype 4 has been found mainly in Asia, including Japan, China, and Taiwan [1, 4]. HEV genotype is one of the important risk factors associated with the disease severity [5–7]. In Cambodia, HEV RNA of genotype 3 has been detected in river water [8] and swine [9]; HEV genotype 1 has also been identified in swine [9]; in patients, anti-HEV IgG and IgM have been found [10, 11]. However, the full-length genome sequence of HEV isolated in Cambodia has not yet been submitted in the DDBJ/EMBL/GenBank database. We have been conducting a seroepidemiological survey on hepatitis virus infections among the general population in Cambodia since 2010 and have reported the seroprevalence and genotype distribution of hepatitis B and C virus among adults in this country [12]. In the current study conducted in collaboration with the Ministry of Health in Cambodia, we investigated the prevalence of HEV infection among the general population in Siem Reap province and sequenced full-length genome of the HEV isolate recovered from an HEV RNA-positive individual.

## Materials and Methods

### Study design

We conducted a cross-sectional study among the general population in Siem Reap province, Cambodia. Based on anticipated anti-HEV IgG rate of 15%, relative precision of 15%, confidence coefficient of 95% and the population size of approximately 3,000 (information from the village/commune chiefs), sample size was calculated to be 755. Therefore, intended sample size was determined to be 800.

### Participants

Seroepidemiological surveys were performed eight times: in February and August, 2010; February and July, 2011; February and August, 2012; June, 2013; and June, 2014 among the general population of Chrey village, Sasar Sdam commune, Krabei Riel commune, and Rohal village in Siem Reap, a province in northwestern Cambodia. The proportion of main activity of general population in Cambodia was 51.8% of employed, 24.7% of student from the data of general population census of Cambodia 2008[13]. Then, we selected Sasar Sdam commune including elementary school according to the characteristic of the population, and three locations which have different background; Chrey was a new urban village, Krabei Riel was an old commune, and Rohal was a sightseeing craft village. On the day of the survey, a duty officer of the Ministry of Health, Cambodia, explained the study protocol to the participants or parents of elementary school students before they were enrolled in the study. The participants who were the residents of Chrey village (333 of total 2034; 16.4%), Krabei Riel commune (189 of total 447; 42.3%), and Rohal village (49 of total 100; 49.0%) were enrolled in this study by the village/commune chiefs. In Sasar Sdam commune, the participants included 240 of total 282 (85.1%) third-year elementary school students (as of 2011) and 57 people living around the school.

### Ethical permission

This study, which was based on questionnaires and blood sample analyses for hepatitis virus infections, was approved by the Ethics Committees for epidemiological research of Hiroshima University, Japan, and the Ministry of Health, Cambodia. Written informed consent was obtained from all the participants or parents of elementary school students. We informed the participants about the results of serological tests for hepatitis virus infections and provided the pamphlet with healthcare information approximately six months later.

### Questionnaires

Questionnaires were used to determine participants’ characteristics such as age, sex, occupation, current health status, current periodic treatment, history of disease or a major injury, history of injection or infusion, operation, blood transfusion, tattoo and holes for pierced earrings.

### Serological tests

About 10 ml of blood drawn from each participant was carefully centrifuged, and the serum samples were transported to Hiroshima University in Japan, where they were tested for hepatitis virus markers to determine the prevalence of hepatitis virus infection. HEV infection was identified by the presence of anti-HEV IgG and IgM antibodies detected using the enzyme immunoassay(EIA) with IgG/IgM anti-HEV EIA (Institute of Immunology Co., Ltd, Tokyo, Japan), and anti-HEV IgA was detected using IMMUNIS IgA anti-HEV EIA (Institute of Immunology). Hepatitis B virus (HBV) was identified based on seropositivity for hepatitis B surface antigen (HBsAg) detected by the reversed passive hemagglutination assay with Mycell II HBsAg (Institute of Immunology), hepatitis B surface antibody (anti-HBs) detected by passive hemagglutination (PHA) using Mycell II anti-HBs (Institute of Immunology) or Chemiluminescence Immunoassay (CLIA) using Architect Osabu (Abbott, Tokyo, Japan), or hepatitis B core antibody (anti-HBc) detected by PHA using Mycell anti-rHBc (Institute of Immunology) or CLIA with Architect HBc II (Abbott, Tokyo, Japan); a sample was considered HBV infection if either HBsAg or anti-HBc were detected with or without anti-HBs. Hepatitis C virus (HCV) infection was confirmed by seropositivity for anti-HCV antibodies by the particle agglutination test using Ortho HCV Ab PA test II (Ortho-Clinical Diagnostics, Inc., Tokyo, Japan). Hepatitis A virus (HAV) infection was detected by CLIA with Architect HAVAB- G (Abbott). HIV infection was determined by the gelatin-particle agglutination test with Serodia HIV-1/2 (Fujirebio Inc., Tokyo, Japan) and the presence of HIV RNA detected by reverse transcription (RT)-PCR [14].

### Detection of HEV RNA

Nucleic acids were extracted from serum samples using Smitest EX-R & D (Medical & Biological Laboratories Co., Ltd. Nagano, Japan). HEV RNA was determined in each anti-HEV IgG-positive sample and in pooled sera of every 10 anti-HEV IgG-negative samples by nested RT-PCR with HE5 primers targeting ORF1 of the HEV genome [15].

### HEV full-length genome sequencing

HEV genomic RNA was reverse transcribed and cDNA was amplified by PCR using primers specific for 12 overlapping regions in the HEV genome (Table 1). Reverse transcription and first-round PCR were conducted using the PrimeScript II High Fidelity One Step RT-PCR Kit (Takara Bio, Inc., Shiga, Japan); second-round PCR was conducted using PrimeSTAR GXL DNA Polymerase (Takara Bio, Inc.). The 3′-Full RACE Core Set (Takara Bio, Inc.) was used to amplify core 3′ sequences. Final products were sequenced using a 3730xl DNA sequencer and the BigDye Terminator v3.1 Cycle Sequencing Kit (Applied Biosystems, Foster City, CA, USA).

**Table 1:** Hepatitis E Virus-specific oligonucleotide primers used in this study.

Primers	Stage-polarity	Nucleotide sequence (5'-3')
Primer set A	1st sense	GCAGACCACGTATGTGGTCG
	2nd sense	CCACGTATGTGGTCGACGCC
	1st antisense	ATRGACACATCATGRTTRTA
	2nd antisense	CCGGCACTRGARTCNCCCTC
Primer set B	1st sense	GCGGARGCNATGGCYCGYCA
	2nd sense	GGCATGACYCGGYTSTAYGC
	1st antisense	TARTCACGSCCRGAYTTYTC
	2nd antisense	CARCTRTARAGGCGYGTTAT
Primer set C	1st sense	AAGTCNACATTTCAYGCCGT
	2nd sense	GTGCAYATATGGGAYAGGCT
	1st antisense	CCTCCRATRAGRGARTGCCG
	2nd antisense	TGRACRCAGCGRTTRAACCT
Primer set D	1st sense	AYTWTGGGYAATAARACYTT
	2nd sense	CCCAGYTRGAGGYCAAYGG
	1st antisense	TTRGAYGCATTRACCAGCCA
	2nd antisense	CCRTCAGGRTARGTRTGRAG
Primer set E	1st sense	CTAAYCCYTTYTGYGGKGA
	2nd sense	CTYTAYACYCGSACYTGGTC
	1st antisense	AGYGANGGGGCCTCRTCRAT
	2nd antisense	GGTGTRTARGCTGCRAACCC
Primer set F	1st sense	AGYTTYGATGCYTGGGARCG
	2nd sense	CCAGCYATAGCYTGGTTYGA
	1st antisense	ATRCCNACCTCRCGRAGRAG
	2nd antisense	GCATCRACMACCACRCAYTT
Primer set G	1st sense	GTYCATGARGCYCARGGYGC
	2nd sense	TTYACTGAGACYACRATTAT
	1st antisense	TTYTCAATAGCRCGRAACCA
	2nd antisense	GTYTTRCTCCAYGCRGATAT
Primer set H	1st sense	AAYGTYACYACCTGYGAGCT
	2nd sense	GAGCTYGTRGAGGCYATGGT
	1st antisense	TGCGAAACAACATCMACACA
	2nd antisense	GCCACATTMGTTARCTTTCGCA
Primer set I	1st sense	AAYACYGTYTGGAAYATGGC
	2nd sense	GGGGATGAYTCTGTTGTRCT
	1st antisense	CGGCGAAGCCCCAGCTGGGG
	2nd antisense	AGCGGCGGGGCGCTGGGAYTG
Primer set J	1st sense	GTGGTTTCTGGGGTGACCGG
	2nd sense	TTCTCAGCCCTTCGCCCTCC
	1st antisense	TTAGTRTARGARTTYACAGG
	2nd antisense	CCATGRATRCARAGCATRAC
Primer set K	1st sense	TCYATYTCYTTYTGGCCYCA
	2nd sense	CCRACGTCYGTNGAYATGAA
	1st antisense	ACAGTRTCAGARACATACAT
	2nd antisense	AGCCARAGYACRTCATTRGC
Primer set L	1st sense	GTCTCAGCCAATGGCGAGC
	2nd sense	GTYGAGAAYGCYCAGCAGGA
	1st antisense	CARAATAAATCAATACTCCCG
	2nd antisense	TACCCACCTTCATYTTRAGACG

### Molecular evolutionary analyses

The number of nucleotide substitutions per site was estimated by a six-parameter modeling method [16], and phylogenetic trees were constructed by the neighbor-joining method [17] based on the number of substitutions. To confirm the credibility of phylogenetic analyses, bootstrap resampling tests were carried out 1,000 times [18]. The analyses were performed using the GENETYX-MAC version 17 software (Genetyx Corporation, Tokyo, Japan).

### Statistical analysis

The data were analyzed using the JMP 10 software (SAS Institute Inc., Cary, NC, USA). Proportions were estimated with the 95% confidence interval (CI); χ^2^ test or Fisher’s exact test and Mantel-extension test for trend were performed to evaluate the difference in the prevalence of viral markers among sex, age, residential, and occupational groups. Univariate analysis using χ^2^ test or Fisher’s exact test and multivariate logistic regression analysis were performed to identify potential risk factors for HEV infection by calculating odds ratios (ORs) and 95% CI. The explanatory variables were sex (reference: woman), age group (reference: 7–19 years old), location (reference: Chrey village), occupation (reference: farmer), and HBV or HCV infection (reference: positive). For all analyses, a *p*-value < 0.05 was considered statistically significant.

## Results

### Characteristics of the participants

Participants’ characteristics are shown in Table 2. In total, there were 868 people, 360 men (41.5%) and 508 women (58.5%); age distribution was from 7 to 90 years (mean, 30.5 ± 18.8; median, 29 years) as of 2014. Among the participants, 38.4% lived in Chrey village, 34.2% in Sasar Sdam commune, 21.8% in Krabei Riel commune, and 5.6% in Rohal village, Siem Reap Province. In terms of occupation, most of the participants were students (40.1%), followed by farmers (33.2%), house workers (7.5%), office workers (6.9%), and craftsmen (2.4%). Other results of the questionnaire are shown in S1 Table.

**Table 2:** Characteristics of participants.

	Characteristics	Total		Men		Women	
		N	(%)	N	(%)	N	(%)
Age group(yr)	7–19	330	(38.0)	146	(40.6)	184	(36.2)
	20–29	118	(13.6)	56	(15.6)	62	(12.2)
	30–39	136	(15.7)	48	(13.3)	88	(17.3)
	40–49	124	(14.3)	61	(16.9)	63	(12.4)
	50–59	85	(9.8)	31	(8.6)	54	(10.6)
	60–90	75	(8.6)	18	(5.0)	57	(11.2)
Location	Chrey village	333	(38.4)	122	(33.9)	211	(41.5)
	Sasar Sdam commune	297	(34.2)	126	(35.0)	171	(33.7)
	Krabei Riel commune	189	(21.8)	70	(19.4)	119	(23.4)
	Rohal village	49	(5.6)	42	(11.7)	7	(1.4)
Occupation	student	348	(40.1)	152	(42.2)	196	(38.6)
	farmer	288	(33.2)	118	(32.8)	170	(33.5)
	house worker	65	(7.5)	1	(0.3)	64	(12.6)
	office worker	60	(6.9)	25	(6.9)	35	(6.9)
	craftsman	21	(2.4)	10	(2.8)	11	(2.2)
	others	86	(9.9)	54	(15.0)	32	(6.3)
Total		868	(100.0)	360	(41.5)	508	(58.5)

### HEV infection prevalence

The results of serologic testing are shown in Table 3. Overall, the prevalence of anti-HEV IgG was 18.4% (160/868; [95% CI: 15.9–21.0%]). Anti-HEV IgG positivity was significantly higher in men than in women (21.9% vs. 15.9%; *p* = 0.0247) and showed statistically significant correlation with older age (*p* < 0.0001). Anti-HEV IgG prevalence differed significantly among the four analyzed locations (*p* < 0.0001). There was also significant difference among occupational/professional groups (*p* < 0.0001) (Table 3).

**Table 3:** Sex-, age-, location- and occupation-specific prevalence of anti-HEV IgG and HEV RNA among the general population in Cambodia.

			anti-HEV IgG positive				HEV RNA positive			
			N	(%)	[95% CI]	*p*-Value	N	(%)	[95% CI]	*p*-Value
Total		868	160	(18.4)	[15.9–21.0]		2	(0.23)	[0–0.55]	
Sex	Man	360	79	(21.9)	[17.7–26.2]	0.0247* ^a^	1	(0.28)	[0–0.82]	0.8065^a^
	Woman	508	81	(15.9)	[12.8–19.1]		1	(0.20)	[0–0.58]	
Age group	7–19	330	19	(5.8)	[3.2–8.3]	<0.0001* ^c^	0	(0.0)	[0–1.1]	0.3839^b^
	20–29	118	25	(21.2)	[13.8–28.6]		0	(0.0)	[0–3.1]	
	30–39	136	32	(23.5)	[16.4–30.7]		1	(0.74)	[0–2.2]	
	40–49	124	36	(29.0)	[21.0–37.0]		1	(0.81)	[0–2.4]	
	50–59	85	30	(35.3)	[25.1–45.5]		0	(0.0)	[0–4.3]	
	60–90	75	18	(24.0)	[14.3–33.7]		0	(0.0)	[0–4.9]	
Location	CV	333	74	(22.2)	[17.8–26.7]	<0.0001* ^a^	1	(0.30)	[0–0.89]	1.0000^b^
	SC	297	29	(9.8)	[6.4–13.1]		1	(0.34)	[0–1.0]	
	KC	189	47	(24.9)	[18.7–31.0]		0	(0.0)	[0–2.0]	
	RV	49	10	(20.4)	[9.1–31.7]		0	(0.0)	[0–7.5]	
Occupation	farmer	288	62	(21.5)	[16.8–26.3]	<0.0001* ^a^	0	(0.0)	[0–1.3]	0.0712^b^
	student	348	24	(6.9)	[4.2–9.6]		0	(0.0)	[0–1.1]	
	house worker	65	21	(32.3)	[20.9–43.7]		1	(1.5)	[0–4.5]	
	office worker	60	18	(30.0)	[18.4–41.6]		0	(0.0)	[0–6.1]	
	craftsman	21	7	(33.3)	[13.2–53.5]		0	(0.0)	[0–17.6]	
	others	86	28	(32.6)	[22.7–42.5]		1	(1.2)	[0–3.4]	

CV: Chrey village, SC: Sasar Sdam commune, KC: Krabei Riel commune, RV: Rohal village, CI: Confidence Interval

* statistically significant variables.

^a^ χ2 test

^b^ Fisher’s exact test

^c^ Mantel-extension test for trend

HEV RNA was detected in two participants (0.23% [0–0.55%]) (Table 3). There were no significant differences in the positivity rate of HEV RNA among sex, age, residential, and occupational groups. Full-length HEV genome could be sequenced for one of the two samples and was classified as genotype 4. Another HEV RNA positive isolate was classified as genotype 3 based on partial ORF1 sequence with HE5 primers [15].

The two HEV RNA-positive participants were analyzed for other hepatitis viruses and HIV (Table 4). They were found positive for anti-HEV IgG and IgM, and one of them was positive for anti-HEV IgA, as well as anti-HBs and anti-HBc, indicating previous HBV infection. Moreover, one of them was positive for anti-HIV antibody, but HIV RNA was not detected.

**Table 4:** HEV RNA positives among the general population in Cambodia.

No	Sex	Age^a^	year	anti-HEV IgG		anti-HEV IgM		anti-HEV IgA		HEV RNA	HEV genotype	HBsAg	anti-HBs	anti-HBc	HBV DNA	anti-HCV	HCV RNA	anti-HAV	anti-HIV	HIV RNA
					COI		COI		COI											
1	M	39	2010	+	23.4	+	1.2	-	0.7	+	4	-	+	+	-	-	-	+	+	-
2	F	33	2011	+	6.8	+	2.3	+	3.3	+	3	-	+	+	-	-	-	+	-	NT

M: Male, F: Female, NT: Not tested, COI: Cut off index

^a^ at survey

### Full-length sequence of the HEV genome

The full-length genome sequence of HEV isolate was recovered from a 39-year-old man at the time of the survey. The isolate designated as CVS-Sie10 (DDBJ/EMBL/GenBank accession number LC042232) had the genome of 7,222 nucleotides (nt) containing three ORFs: ORF1 [1–5,115 nt; 1,705 amino acids (aa)], ORF2 (5,115–7,136 nt; 674 aa), and ORF3 (5,143–5,484 nt; 114 aa), a 3′ UTR, and a poly-A tail.

The alignment of the CVS-Sie10 genome with published HEV genotype 4 sequences showed that this isolate was close to the swine China isolates swGX40, SS19, and swDQ with sequence identities of 95.57%, 94.37%, and 91.94%, respectively (Table 5), and the same length of ORF1, ORF2, and ORF3. CVS-Sie10 displayed weak homology with other HEV genotypes. Thus, it demonstrated 76.99%, 76.83%, 78.09%, and 79.34% identity with the Burma isolate of genotype 1, the Mexico isolate of genotype 2, the HEV-US2 isolate of genotype 3, and the JBOAR135-Shiz09 isolate of genotype 5, respectively.

**Table 5:** Identity of full-genome sequences of HEV isolates known.

Genotype	Isolate name	Accession number	Source		Nucleotides length	Identity with CVS-Sie10 (%)	Amino acids length		
			Country	host			ORF1	ORF2	ORF3
4	CVS-Sie10	LC042232	Cambodia (Siem Reap)	human	7222		1705	674	114
	swGX40	EU676172	China (Guangxi)	swine	7269	95.57	1705	674	114
	SS19	JX855794	China (Guangdong)	swine	7233	94.37	1705	674	114
	swDQ	DQ279091	China	swine	7234	91.94	1705	674	114
	JYK-Tok03C	AB291964	Japan (Tokyo)	human	7244	87.44	1705	674	114
	HEVN2	AB253420	Japan (Okinawa)	human	7253	87.40	1707	674	114
	EChZ20	HM439284	China (eastern China)	human	7229	86.59	1704	674	114
	W2-5	JQ655736	China (Beijing)	human	7261	85.50	1706	674	114
	JYI-ChiSai01C	AB197674	China (Shanghai)	human	7260	85.00	1706	674	114
1	Burma	M73218	Burma	human	7207	76.99	1693	660	123
2	Mexico	M74506	Mexico	human	7180	76.83	1691	659	123
3	HEV-US2	AF060669	the United States	human	7277	78.09	1708	660	122
5	JBOAR135-Shiz09	AB573435	Japan (Shizuoka)	wild boar	7267	79.34	1708	674	112

**Fig 1:**
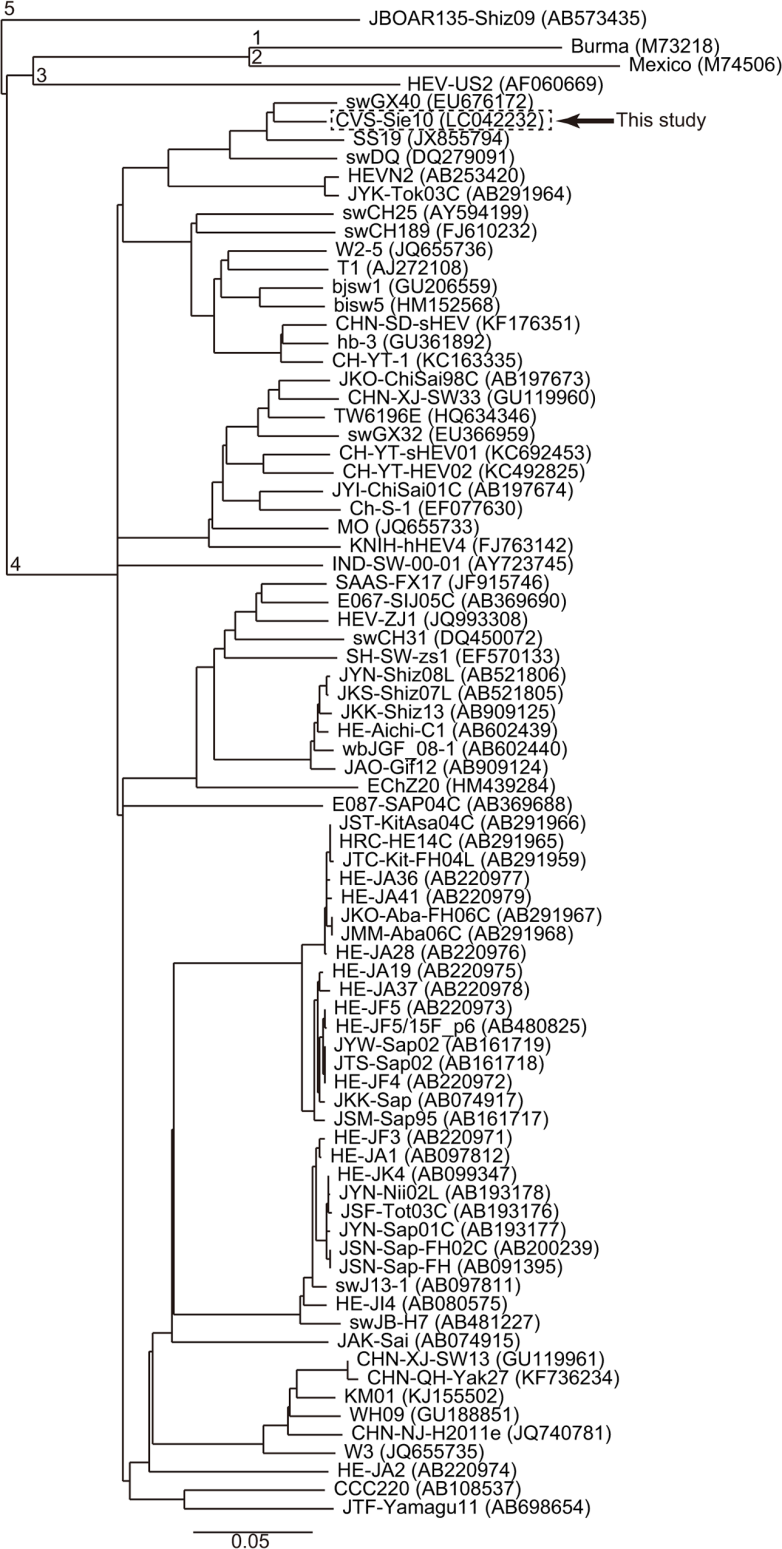
Phylogenetic tree constructed based on HEV full-length genomes using the neighbor-joining method. Each of HEV genotypes 1, 2, 3, and 5 is represented by a single isolate, while for genotype 4, all the isolates with reported complete or near-complete genome sequence are presented. GenBank accession numbers are shown in parentheses; scale bar indicates nucleotide substitutions per site.

Based on full-length genome sequences of the CVS-Sie10 and other HEV isolates, we constructed a phylogenetic tree (Fig 1), which showed that CVS-Sie10 clustered on a branch separate from the other genotype 4 sequences, and close to the China isolates swGX40, SS19, and swDQ.

### Potential risk factors for HEV infection

The odds ratios and *p*-values of potential risk factors for anti-HEV IgG positivity in univariate analysis and multivariate logistic regression model are shown in Table 6. In multivariate analysis, men (AOR: 1.9 [1.2–2.8] *p* = 0.0025) and older age (if the 7–19-year-old population group is taken as baseline) were significantly associated with anti-HEV IgG seropositivity. There were no significant differences among the four locations. The odds ratio of anti-HEV IgG seropositivity for house workers was more than twice higher than that for farmers (AOR: 2.3 [1.2–4.5]; *p* = 0.0109), and that for others was significantly higher than farmers (AOR: 1.8 [1.0–3.2]; *p* = 0.0464). On the other hand, HBV and HCV infections were not associated with anti-HEV IgG seropositivity.

**Table 6:** Univariate and multivariate analysis of positivity for anti-HEV IgG among the general population in Cambodia.

			anti-HEV IgG					
			Univariate analysis^a^			Multivariate analysis^b^		
		N	OR	[95% CI]	*p*-Value	AOR	[95% CI]	*p*-Value
Sex	Man	360	1.5	[1.1–2.1]	0.0247*	1.9	[1.2–2.8]	0.0025*
	Woman	508	1			1		
Age group(yr)	7–19	330	1			1		
	20–29	118	4.4	[2.3–8.3]	<0.0001*	5.7	[1.7–17.8]	0.0037*
	30–39	136	5.0	[2.7–9.3]	<0.0001*	7.1	[1.8–26.2]	0.0038*
	40–49	124	6.7	[3.7–12.3]	<0.0001*	9.2	[2.4–34.1]	0.0010*
	50–59	85	8.9	[4.7–17.0]	<0.0001*	12.3	[3.1–46.3]	0.0002*
	60–90	75	5.2	[2.6–10.4]	<0.0001*	6.7	[1.6–26.3]	0.0068*
Location	CV	333	1			1		
	SC	297	0.38	[0.24–0.60]	<0.0001*	0.95	[0.51–1.8]	0.8708
	KC	189	1.2	[0.76–1.8]	0.4912	1.2	[0.71–1.9]	0.5667
	RV	49	0.90	[0.43–1.9]	0.7747	0.56	[0.23–1.2]	0.1716
Occupation	farmer	288	1			1		
	student	348	0.27	[0.16–0.45]	<0.0001*	1.9	[0.58–5.8]	0.2643
	house worker	65	1.7	[0.96–3.1]	0.0642	2.3	[1.2–4.5]	0.0109*
	office worker	60	1.6	[0.84–2.9]	0.1559	1.5	[0.75–3.1]	0.2231
	craftsman	21	1.8	[0.71–4.7]	0.2098	2.5	[0.87–6.8]	0.0742
	others	86	1.8	[1.0–3.0]	0.0357*	1.8	[1.0–3.2]	0.0464*
HBV infection	positive	247	2.0	[1.4–2.9]	<0.0001*	1.1	[0.75–1.7]	0.5593
	negative	621	1			1		
HCV infection	positive	34	1.9	[0.89–4.1]	0.0921	1.2	[0.52–2.7]	0.6224
	negative	834	1			1		

OR: Odds Ratio, AOR: Adjusted Odds Ratio, CI: Confidence Interval, CV:Chrey village, SC: Sasar Sdam commune, KC: Krabei Riel commune, RV: Rohal village

^a^ χ^2^ test or Fisher’s exact test

^b^ Logistic regression analysis: R^2^ = 0.1113, Model p<0.0001*, N = 868

* statistically significant variables

## Discussion

In this study, we performed a seroepidemiological survey for HEV infection in Cambodia. As a result, we identified the first human case of HEV genotype 4 in Cambodia and performed full-length genome sequencing of the isolate. One case of HEV genotype 3 was also detected. In developing countries, HEV infection is usually caused by genotype 1 viruses [1]; however, the data obtained in our study shows different genotype. In previous studies in Cambodia, HEV genotype 3 was detected in river water [8], and genotypes 1 and 3 were recovered from swine [9]. Partial sequences of HEV genotype 1 (accession number: DQ145797) and genotype 3 (accession number: DQ145792) from humans have been determined, but genotype 4 has not been reported. In this study, we sequenced, for the first time, the full-length genome of human HEV genotype 4 isolated in Cambodia.

Two HEV RNA-carrying participants were also positive for anti-HEV IgM and had a prior history of HBV infection as evidenced by the presence of anti-HBs and anti-HBc antibodies; one of the participants was also anti-HIV-positive. The detection of anti-HEV IgM indicates that both individuals had early stages of HEV infection. HEV genotype 4 has been reported to cause higher rate of aggravation from viral infection than genotype 3 [5, 6, 19], however, according to their answers to our questionnaire, both participants (genotype 3 and genotype 4) did not receive periodic treatment in hospitals and did not have serious problems with health. Moreover, they were confirmed asymptomatic during the interview 6 months after a blood test. Both of them had a prior history of HBV infection, which is not a rare case for Cambodia, where adult population has high anti-HBc positive rate of 38.5% [12]. Our multivariate analysis revealed no association between HEV and HBV infections.

Aggravation due to HEV infection is infrequent, and was not observed in this study. HEV genotype 4 is more likely to cause aggravation, and sometimes cause death [20, 21]; in recent years, the risk of HEV triggering chronic hepatitis in immunocompromised patients, including HIV carriers [22] and organ transplant recipients [23, 24] has been reported. These data underscore the importance of investigating the prevalence and performing genetic analysis of HEV infection in Cambodia, which is the part of Asia believed to be heavily affected by HEV infection, and the necessity of developing preventive measures against HEV spread.

In our survey, the overall rate of anti-HEV IgG positivity among 868 participants was 18.4%. There has been no prior study on the prevalence of HEV infection in the general population in Cambodia; however, the rate of anti-HEV IgG positivity in patients with high aspartate transaminase and alanine transaminase levels was determined as 5.5% [10], and the rate of anti-HEV IgM positivity in feverish patients was 11.1% [11]. Other studies have indicated that the rate of anti-HEV IgG positivity among blood donors in Japan was 3.4% [25] and in the general population of the Mekong River basin in Vietnam it was 9% [26]; among the general population of the East China Sea coast in the same country it was 28.1% (143/509 [95% CI: 24.2–32.0%]) (our unpublished data). In China, Taiwan, India, and Thailand, HEV IgG positivity rate was 20.2%, 10.7%,4.0%, and 2.8–7%, respectively, while in urban and rural Malaysia, it was 2% and 44–50%, respectively [27]. Compared to these data, the rate of anti-HEV IgG positivity detected in this study is slightly higher than average. There might be possible selection bias because we could not perform the random sampling. However, the screening for hepatitis virus infections does not conducted among the general population in Cambodia. On the other hand, the sensitivity of detection of anti-HEV IgG differs according to the assay. We detected by the kit used the purified recombinant HEV ORF2 protein in EIA same as the method described previously [28]. The results of the detection of anti-HEV IgG using this recombinant HEV ORF2 protein showed that the positivity of anti-HEV IgG was as high as 98% among the totally 57 acute hepatitis samples with positive for HEV RNA (56/57 samples: anti-HEV IgM/IgG +/+, HEV RNA +) [28–30](unpublished data). Furthermore, the sensitivity of the assay was not depend on genotype, and it also showed the high positivity among the swine [31]. Therefore, we considered that the sensitivity of detection of anti-HEV IgG by our used assay was high.

Our present results demonstrate that men have a significantly higher rate of HEV infection than women; similar association has been observed in Japan among blood donors [25], and reported by the National Epidemiological Surveillance of Infectious Diseases [32]. Moreover, similar correlation has been shown in the United Kingdom [33, 34], the United States [35], and China [36]. The reason for such an association is not clear; given that HEV infection is primarily transmitted via the oral route, it can be hypothesized that game meat consumption and other food preferences may be involved.

Higher prevalence of anti-HEV IgG in older age groups indicates age-related risk. Even after the adjustment for other factors, older people demonstrate significantly higher rates of anti-HEV IgG positivity, if the 7–19-year-old population group is taken as baseline. In Vietnam [26] and Indonesia [37], it has been reported that the rate of anti-HEV positivity rises with age. Conceivable reason for the correlation of anti-HEV IgG positivity with age is because currently occurring new infections cause the number of people with a prior history of HEV infection to increase with age. Although the period when HEV RNA is detectable in the blood after HEV infection is brief constituting 28.3 days [38], two HEV RNA-positive participants (0.23%: 2/868 [95%CI: 0–0.55%]) were identified in our cross-sectional study of 868 people. Assuming that HEV RNA detection period is about four weeks, HEV incidence is estimated at 3.00/100 person-years (0–7.2/100 person-years). Among rural Chinese population, the rate of anti-HEV IgG positivity was 38%, with the incidence of 2.8/10,000 person-years [36], and in rural Bangladesh regarded as HEV endemic region, the prevalence of anti-HEV total Ig was 22.5% and the incidence was 63.9/1,000 person-years [39]. Compared to these data, HEV incidence determined in our study can be regarded as high, indicating that infection control measures are required.

HEV infection is often foodborne in developed countries, including Japan and Europe [4, 6]; in developing countries, contaminated water is considered to be a major cause [40–42]. Thus, there has been an outbreak of HEV infection in southwestern Vietnam along the Hau river, adjacent to Cambodia [43], and HEV has been detected in the water of Siem Reap River, Cambodia [8], indicating a possibility that HEV outbreak could also occur in Cambodia. In turn, HAV positive rate among the participants in this study was 88.8% (767/864; [86.7–90.9%]): adults (18 years or older) had 99.6% (552/554; [99.1–100.0%]) and minors (17 years or younger) had 69.4% (215/310; [64.2–74.5%]), indicating age-related effect.

In Cambodia, nearly everyone is assumed to have HAV exposure by the time they reach adulthood. As with HEV infection, HAV is transmitted via the fecal-oral route, and by adulthood, nearly everyone is HAV-infected via contaminated food or water, indicating serious issues with health management. To prevent HEV infection in Cambodia, proper water hygiene is regarded as the first necessary measure. Multivariate analysis also showed that house workers had a significantly higher rate of anti-HEV IgG positivity than farmers, office workers, students, and craftsmen, which suggested possible problems with water hygiene for cooking or washing the cloth, or handling with raw stuff [44].

The HEV isolate fully sequenced in this study is most closely related to the strains isolated from swine in Guangxi and Guangdong, both in southern China. The cause of HEV infection for the participants with detected HEV RNA (including the CVS-Sie10 isolate) is unknown; however, HEV is a zoonotic pathogen that infects pigs, wild boars, and other animals, and causal relationship between consumption of contaminated meat and hepatitis E onset has been confirmed [29]. In Japan, the majority of foodborne infections are presumed to be related to meat consumption [32], which can be also true in Cambodia; therefore, it is possible that the consumption of undercooked meat or drinking water contaminated by animal waste may be the cause of HEV infection for the participants in this study.

In Japan, HEV infection by blood transfusion has been reported in Hokkaido [45, 46], which is regarded as a region with increasing danger of HEV infection; therefore, it is the only place in the world where donor blood is screened for HEV RNA. In recent years, HEV infection through blood transfusion has been regarded as a growing problem also in the West, and the pros and cons of HEV blood screening have been considered. In this study, we identified HEV genotype 4 characterized with high post-infection aggravation rate, and also found that new HEV infections occur very frequently in Cambodia, raising concerns about HEV infection through blood transfusion and suggesting that it may be necessary to enhance the safety of blood supply.

The present study is the first to detect HEV genotype 4 in human blood in Cambodia and to report sequencing of genotype 4 full-length genome. Our survey revealed high HEV prevalence among Cambodian general population, including frequent cases of early HEV infection, suggesting that measures to prevent HEV infection, such as improving water and food safety and spreading health and hygiene education in school, are urgently required.

## Supporting Information

S1 Table Results of the questionnaire. This table shows the answers of eight questions. Question 3 and 4 were not asked to elementary school students.
(DOCX)
