# Supplementary material for: Hepatitis E Virus in Cambodia: Prevalence among the General Population and Complete Genome Sequence of Genotype 4
Source: PLoS One. 2015 Aug 28;10(8):e0136903. doi: 10.1371/journal.pone.0136903 (PMC4552640; doi:10.1371/journal.pone.0136903)
Supplement: S1 Table — This table shows the answers of eight questions. Question 3 and 4 were not asked to elementary school students. (DOCX) [file pone.0136903.s001.docx]

**S1 Table. Results of the questionnaire**

| **Questionnaire** |  | **N** | **(%)** |
| --- | --- | --- | --- |
| Q1. “Are you healthy now?” | Yes | 428 | (49.3) |
|  | No | 436 | (50.2) |
|  | Unknown | 4 | (0.5) |
| Q2. “Do you receive periodic treatment in hospitals now? | Yes | 299 | (34.4) |
|  | No | 552 | (63.6) |
|  | Unknown | 17 | (2.0) |
| Q3. “Have you had a disease or a major injury in your life?” | Yes | 250 | (28.8) |
|  | No | 376 | (43.3) |
|  | Unknown | 242 | (27.9) |
| Q4. “Have you received treatment by injection or infusion in your life?” | Yes | 479 | (55.2) |
|  | No | 144 | (16.6) |
|  | Unknown | 245 | (28.2) |
| Q5. “Have you had an operation in your life?” | Yes | 68 | (7.8) |
|  | No | 790 | (91.0) |
|  | Unknown | 10 | (1.2) |
| Q6. “Have you had a blood transfusion in your life?" | Yes | 12 | (1.4) |
|  | No | 772 | (88.9) |
|  | Unknown | 84 | (9.7) |
| Q7. “Do you have a tattoo in your body? | Yes | 55 | (6.3) |
|  | No | 691 | (79.6) |
|  | Unknown | 122 | (14.1) |
| Q8. “Do you have any holes for pierced earrings in your body?” | Yes | 458 | (52.8) |
|  | No | 311 | (35.8) |
|  | Unknown | 99 | (11.4) |

This table shows the answers of eight questions. Question 3 and 4 were not asked to elementary school students.
